# Book Notices

**Published:** 1879-03-20

**Authors:** 


### *BOOK NOTICES.*

CONCLUSIONS OF THE BOARD OF EXPERTS AUTHORIZED BY CONGRESS TO INVESTIGATE THE YELLOW FEVER EPIDEMIC OF 1878—*Being a Reply to Questions of the Committee of the Senate and House of Representatives upon the Subject of Epidemic Diseases.*

In this we have a pamphlet of 36 pages containing 90 propositions upon which the Committee seem to have united, with one exception, Dr. Falligant, who differs on certain points, particularly in regard to the importation theory; it being held by the majority that the disease has invariably come from the West Indies.

The Congressional Board of Experts continued the investigation which had been instituted by the Yellow Fever Commission, in October last. The Board assembled and organized at Memphis in the month of December, and uniting with the previous Yellow Fever Commission, continued their observations until the number of places examined amounted to about fifty. They completed their work and convened in Washington City on January 15th, and agreed upon the report above refered to.

Whatever Congress or the public may have anticipated, the Medical Profession in the United States did not look for any definite or satisfactory solution of the mysteries connected with the origin, nature, etc., of yellow fever. The time for the investigation was too short, if indeed it were possible in the present state of medical science, to make the discoveries sought for. The result has been ninety propositions embodying the views—theoretical in the main—of the intelligent gentlemen constituting this Board.

We find in these propositions nothing specially new, yet the Board has done all that is perhaps attainable with the lights before them. The Report should be read as containing useful historical information in regard to yellow fever, and a summary of probably the latest views and conclusions upon the subject, and also in regard to cholera, upon which they were also instructed to report.

Among the recommendations submitted to Congress we find that of stationing Medical Health Officers at various foreign ports, where yellow fever, cholera and other infectious epidemics prevail, enjoined to keep watch and report the origin or prevalence of such diseases, and to co-operate in such national quarantine establishments and operations as now exist or may be established. The plan suggested embraces also medical officers for home service.

DIPHTHERIA, ITS NATURE AND TREATMENT, VARIETIES AND LOCAL EXPRESSIONS, by MORELL MACKENZIE, M.D., London; senior physician to the Hospital for Diseases of the Throat and Chest, etc., etc.—Philadelphia, Lindsay & Blackeston, 1879.

This is a well-written, instructive and practical treatise of 100 pages, in which we have presented the history, etiology, diagnosis, pathology and treatment of diphtheria. It is well worthy of perusal by every practitioner.

CONSPECTUS OF ORGANIC MATERIA MEDICA AND PHARMACAL BOTANY, comprising the *Vegetable and Animal Drugs*, their physical character, geographical origin, classification, constituents, doses, adulterations, etc.; table of the tests and solubilities of the alkaloids appended; by L. E. Sayre, Ph. G.—Philadelphia, D. G. Brinton, 115 S. Seventh street.

The above is a neatly gotten up work of 210 pages, embracing a department of Materia Medica which has been much neglected. Such a work has been long needed and will at once be welcomed by every Botanist and every student of medicine.

LECTURE ON FRACTURE OF THE FEMUR, by EDWARD BORCK, M.D., member of the Medical and Chirurgical Faculty, of Maryland, Baltimore Med. Association, St. Louis Med. Society. Missouri State Med. Association, Tri-State Med. Society; formerly Assistant Surgeon to West Building Hospital, Baltimore, etc.; with illustrations.—Saint-Louis, Geo. O. Rumbold & Co., 1879.

The above is a very interesting and practical monograph on the important subject of fracture of the femur. It will well repay perusal.

TRANSACTIONS OF THE MEDICAL SOCIETY OF THE STATE OF PENNSYLVANIA, AT ITS 28th ANNUAL SESSION HELD AT PITTSBURG, MAY, 1878.

We regret that we have not space at present to give an extended review of this very creditable volume. It is the largest and most interesting volume of transactions that we remember to have seen from any State society. A great many interesting papers and reports, so varied and important that we will not undertake to specify any one of them, but advise our readers to procure a copy for perusal. We expect to refer to the contents of this work at a future time.

Published by the Society; Collins, printer, Philadelphia.

TRANSACTIONS OF THE MEDICAL SOCIETY OF THE STATE OF TENNESSEE.—April, 1878.

Will notice hereafter.

